# Calcitriol, the Bioactive Metabolite of Vitamin D, Increases Ventricular K^+^ Currents in Isolated Mouse Cardiomyocytes

**DOI:** 10.3389/fphys.2018.01186

**Published:** 2018-08-24

**Authors:** María Tamayo, Laura Martin-Nunes, Almudena Val-Blasco, Maria J. Piedras, María J. Larriba, Nieves Gómez-Hurtado, María Fernández-Velasco, Carmen Delgado

**Affiliations:** ^1^Biomedical Research Institute “Alberto Sols” CSIC-UAM/CIBER-CV, Madrid, Spain; ^2^Innate Immune Response Group, IdiPAZ/CIBER-CV, La Paz University Hospital, Madrid, Spain; ^3^University Francisco de Vitoria, Madrid, Spain; ^4^Biomedical Research Institute “Alberto Sols” CSIC-UAM/CIBERONC, Madrid, Spain

**Keywords:** calcitriol, potassium currents, cardiomyocytes, vitamin D, cellular electrophysiology, ionic channel remodeling, Akt

## Abstract

Calcitriol, the bioactive metabolite of vitamin D, interacts with the ubiquitously expressed nuclear vitamin D receptor (VDR) to induce genomic effects, but it can also elicit rapid responses via membrane-associated VDR through mechanisms that are poorly understood. The down-regulation of K^+^ currents is the main origin of electrophysiological remodeling in pathological hypertrophy and heart failure (HF), which can contribute to action potential prolongation and subsequently increase the risk of triggered arrhythmias. Adult mouse ventricular myocytes were isolated and treated with 10 nM calcitriol or vehicle for 15–30 min. In some experiments, cardiomyocytes were pretreated with the Akt inhibitor triciribine. In the adult mouse ventricle, outward K^+^ currents involved in cardiac repolarization are comprised of three components: the fast transient outward current (I_tof_), the ultrarapid delayed rectifier K^+^ current (I_kur_), and the non-inactivating steady-state outward current (I_ss_). K^+^ currents were investigated using the whole-cell or the perforated patch-clamp technique and normalized to cell capacitance to obtain current densities. Calcitriol treatment of cardiomyocytes induced an increase in the density of I_tof_ and I_kur_, which was lost in myocytes isolated from VDR-knockout mice. In addition, calcitriol activated Akt in cardiomyocytes and pretreatment with triciribine prevented the calcitriol-induced increase of outward K^+^ currents. In conclusion, we demonstrate that calcitriol via VDR and Akt increases both I_tof_ and I_kur_ densities in mouse ventricular cardiomyocytes. Our findings may provide new mechanistics clues for the cardioprotective role of this hormone in the heart.

## Introduction

Cardiac remodeling is the response of the heart to various damaging stimuli, such as long standing hypertension or myocardial infarction. This physiological process is characterized by the triggering of “compensatory" pathways such as, the renin-angiotensin system (RAS) and the sympathetic nervous system (SNS). Initially, this mechanism is beneficial but sustained activation of neurohormonal systems can cause additional damage to the heart, commonly in the form of hypertrophy, apoptosis and fibrosis, contributing to the adverse remodeling process, and accelerating the transition toward heart failure (HF) (Heusch et al., 2014; Hartupee and Mann, 2017). Moreover, ventricular remodeling modifies the functioning of ion channels and transporters such as that cardiac rhythm disturbances occur, which is termed “arrhythmogenic remodeling" (Nattel et al., 2007). K^+^ currents play key roles in shaping the action potentials (APs), and adverse ventricular remodeling induces down-regulation of several K^+^ currents, which are associated with prolonged QT intervals, increased risk of malignant arrhythmias and sudden cardiac death. It is well known that almost one-half of HF patients die from sudden cardiac death, most probably due to ventricular arrhythmias (Nass et al., 2008; Lehnart et al., 2009).

Vitamin D deficiency is highly prevalent worldwide, an in particular among the elderly population (Holick, 2007). Low levels of vitamin D have been associated with worse outcomes in patients with HF (Liu et al., 2011; Gotsman et al., 2012) and vitamin D supplementation has been shown to ameliorate symptoms and decrease mortality (Gotsman et al., 2012; Witte et al., 2016). The biologically active form of vitamin D is 1,25-dihydroxycholecalciferol or calcitriol, which translocates the cell membrane and cytoplasm to reached the nucleus of the cell, where it binds to vitamin D receptor (VDR). In turn, VDR can bind to retinoic X receptor to serve as a nuclear transcription factors, regulating the expression of numerous genes. In addition to these genomic responses, calcitriol can mediate rapid responses by binding to cell membrane VDR, interacting with ion channels (Menegaz et al., 2011; Zanatta et al., 2012; Tamayo et al., 2017) and membrane-based signaling pathways (Larriba et al., 2014).

Previous reports have postulated that activation of the PI3K pathway can modulate cardiac K^+^ channels in cardiomyocytes (Yang et al., 2012; Gomez-Hurtado et al., 2014) and Akt is a well-known target of PI3K, activated by calcitriol in other cellular systems.

Calcitriol has been demonstrated to initiate a VDR-dependent rapid activation of PI3K/Akt signaling in osteoblasts (Vertino et al., 2005). In addition, the anti-apoptotic properties of calcitriol in osteoblasts are thought to be related to the non-genomic activation of a VDR/PI3K/Akt pathway (Zhang and Zanello, 2008). Moreover, activation of Akt by calcitriol has also been observed in leukemia, squamous cell carcinoma and skeletal muscle cells (Ma et al., 2006; Zhang et al., 2006; Buitrago et al., 2012).

Vitamin D receptor has been identified in numerous cardiovascular cell types including cardiomyocytes (Tishkoff et al., 2008; Norman and Powell, 2014). Whereas experimental studies have demonstrated rapid effects of calcitriol on cardiac calcium channels (Zanatta et al., 2012; Tamayo et al., 2017), its effects on cardiac potassium channels remain elusive. Thus, in the present study we used patch-clamp recordings to examine the rapid responses of calcitriol on the main K^+^ currents responsible for cardiac repolarization in the mouse and the potential mechanism (s) involved. Our results show that calcitriol increases outward K^+^ current densities in ventricular myocytes via Akt signaling, and that this mechanism can contribute to the protective effect of vitamin D on the heart.

## Materials and Methods

### Animals

Adult (2–3 months old) male C57BL/6J mice and VDR knockout (KO) mice were originally generated by Dr. Marie Demay (Harvard Medical School, Boston, MA, United States) (Li et al., 1997) and kindly donated by Dr Alberto Muñoz (Biomedical Research Institute “Alberto Sols” CSIC-UAM, Madrid, Spain) were used. All experiments on mice were performed after approval by the Bioethical Committee of the *Consejo Superior de Investigaciones Cientificas* following recommendations of the Spanish Animal Care and Use Committee (Proex 035-15) according to the guidelines for ethical care of experimental animals of the European Union (2010/63/EU).

### Electrophysiological Procedures and Data Analysis

Adult mouse ventricular cardiomyocytes were isolated as described (Delgado et al., 2015). Single rod-shaped, Ca^2+^-tolerant myocytes were treated with calcitriol or vehicle for 15–30 min and used for electrophysiological recordings within 4 h of isolation.

The electrophysiological protocols used to record APs and K^+^ currents in this study were the same as those previously described (Trépanier-Boulay et al., 2001; Brouillette et al., 2004; Gómez-Hurtado et al., 2017). Briefly, ventricular myocytes were placed in a chamber mounted on the stage of an inverted microscope and allowed to adhere for 5 min before being superfused with Tyrode’s solution. Whole-cell voltage-clamp and current clamp recordings were obtained in the ruptured patch configuration using an Axopatch 200B patch clamp amplifier (Molecular Devices, Sunnyvale, CA, United States). The patch pipette resistance was 1.0–2 MΩ and the pipette was filled with a solution containing (in mM): 135 KCl, 4 MgCl_2_, 5 EGTA, 10 HEPES, 10 glucose, 5 Na_2_ATP, and 5 disodium creatine phosphate; the pH was adjusted to 7.2 with KOH. Whole-cell voltage clamp experiments were performed at room temperature (24–26°C), whereas whole-cell current clamp experiments (AP recordings) were carried out at 36–37°C.

The external solution for K^+^ current recordings contained (in mM): 135 NaCl, 10 glucose, 10 HEPES, 1 MgCl_2_, 1 CaCl_2_, 5.4 KCl, and 2 CoCl_2_; the pH was adjusted to 7.4 with NaOH. The external solution for AP recordings contained (in mM): 140 NaCl, 10 glucose, 10 HEPES, 1.1 MgCl_2_, 1.8 CaCl_2_, and 4 KCl; the pH was adjusted to 7.2 with NaOH.

Current density was calculated from the current amplitude normalized to the membrane capacitance. Membrane capacitance (C_m_) was elicited by applying ±10 mV voltage steps from −60 mV and C_m_ was calculated according to the following equation:

$${\text{C}}_{\text{m}}=\tau_{\text{c}}{\text{I}}_{0}/\Delta {\text{V}}_{\text{m}}[1-({\text{I}}_{\infty }/{\text{I}}_{0})]$$

where τ_c_ is the time constant of the membrane capacitance, I_0_ the maximum capacitance current value, ΔV_m_ the amplitude of the voltage step, and I∞ the amplitude of the steady-state current.

In some experiments the perforated patch-clamp technique was used to record the time course of the effects of calcitriol on total K^+^ currents. Perforated-patch recordings were started 5–25 min after a giga-seal was obtained. Just before use, the tip of the pipette was filled with antibiotic-free intracellular solution and the remainder of the pipette was backfilled with pipette solution containing 250 μg/mL amphotericin B. Depolarizing pulses from a holding potential of −60 to −40 mV were used to monitor the access resistance. Recordings of I_total_ were initiated when the access resistance was stabilized at 6–13 MΩ. I_total_ was elicited by depolarizing pulses to +50 mV from a holding potential of −80 mV every 10 s. The extracellular solution for I_total_ recordings for perforated-patch experiments was the same that we used for the whole-cell experiments. The intracellular solution used to elicit recordings in the perforated-patch configuration contained (in mM): 110 potassium aspartate, 20 KCl, 1 EGTA, 1 CaCl_2_, 5 HEPES, 1 MgCl_2_, 250 μg/mL amphotericin B; pH adjusted to 7.2 with KOH.

### Western Blotting

Cardiomyocytes were homogenized in a buffer containing 320 mM sucrose, 50 mM Tris and 0.2% nonidet P-40, supplemented with a phosphatase, trypsin and protease inhibitor cocktail (Sigma-Aldrich, Madrid, Spain). Lysed cells were centrifuged at 13,000 rpm for 10 min at 4°C and the supernatants were used for immunoblotting. Equal amounts of protein were separated on 10% SDS–PAGE gels and transferred to polyvinylidene difluoride membranes. The membranes were blocked with 5% non-fat milk and incubated overnight with the following primary antibodies: phospho-Akt (Thr308) and Akt (Cell Signaling Technology, Danvers, MA, United States)., blots were incubated with peroxidase-conjugated secondary antibodies and immunoreactive bands were detected using the Amersham ECL^TM^ protein detection system (GE Healthcare, Piscataway, NJ, United States) and medical x-ray film blue (Agfa HealthCare, Morstel, Belgium). Films were scanned with a NIKON camera and quantified by ImageJ software (NIH, Bethesda, MD, United States).

### Reagents

The Akt inhibitor triciribine, calcitriol and amphotericin B were purchased from Sigma-Aldrich Co. (St. Louis, MO, United States). A stock solution of calcitriol was prepared using DMSO as vehicle (<0.01% DMSO).

### Statistical Analysis

Data are presented as means ± SEM. Statistical analysis was performed with GraphPad Prism 5.0 (GraphPad Software Inc., La Jolla, CA, United States). Statistical significance was evaluated using unpaired or paired Student’s *t-*test when appropriate. A value of *P* < 0.05 was considered statistically significant.

## Results

### Calcitriol Increases Outward K^+^ Currents

**FIGURE 1:**
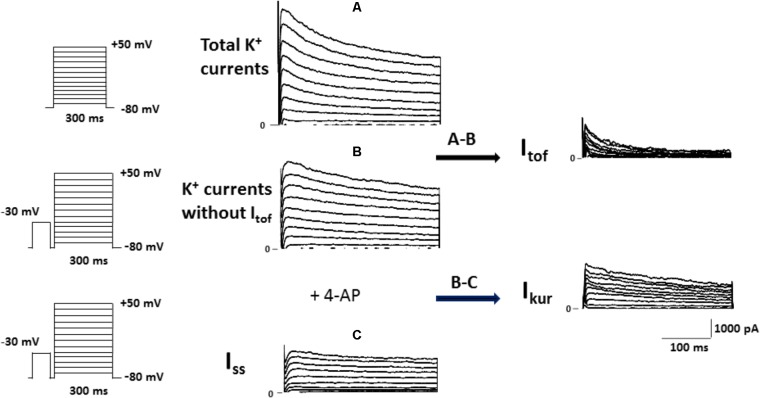
Protocol used to isolate the fast transient outward K^+^ current (I_tof_), the ultrarapid delayed rectifier K^+^ current (I_kur_) and the non-inactivating steady-state outward current (I_ss_) in mouse ventricular cardiomyocytes. **(A)** Superimposed current records of total K^+^ currents activated by voltage protocol shown on the left. **(B)** Family of K^+^ currents obtained from the same cell as in **(A)** (C_m_ = 212 pF), but voltage-clamp steps were each preceded by an inactivating pulse at −30 mV, shown in the left panel. I_tof_ was then calculated by substracting the current recording with **(A)** and without **(B)** the inactivating prepulse. **(C)** Superimposed current records of K^+^ currents obtained after the same cell as those in **(B)** (C_m_ = 212 pF) was perfused with 4-AP, which selectively blocks I_Kur_. The current remaining after this protocol is I_ss_, and I_kur_ was then obtained by subtracting the current in the absence and in the presence of 4-AP.

**FIGURE 2:**
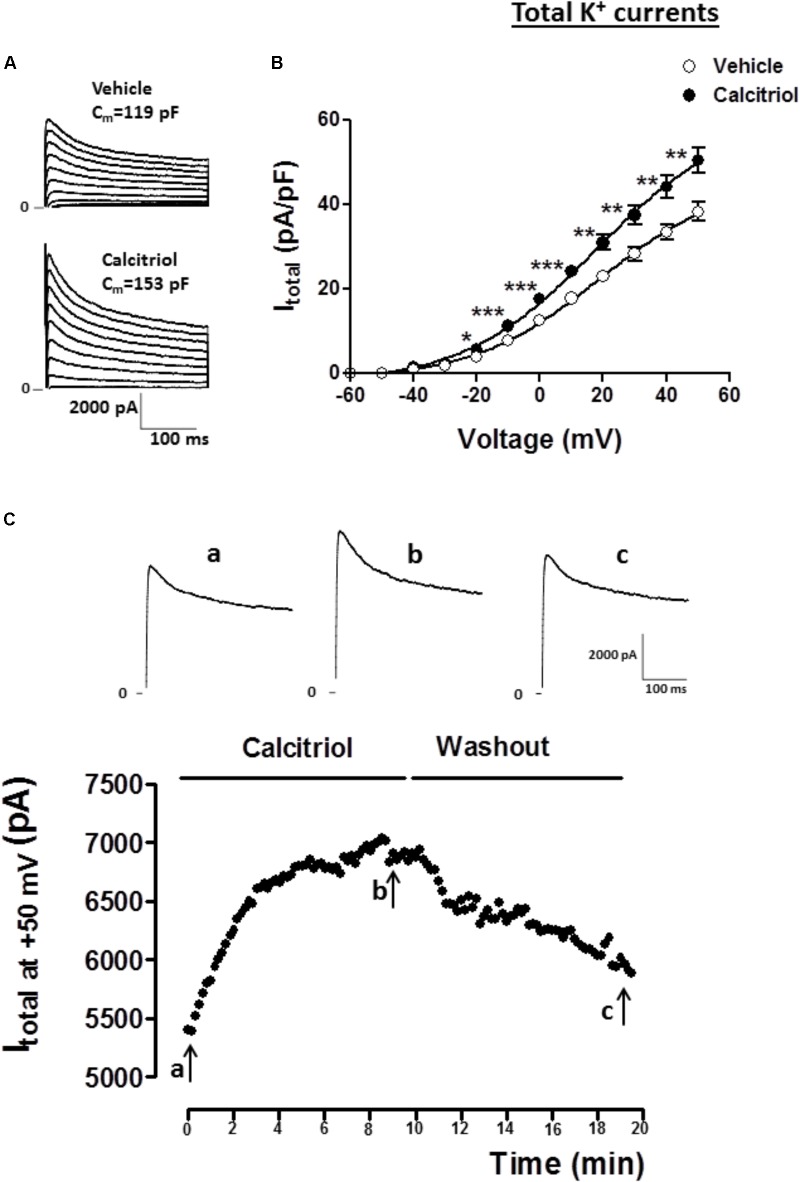
Calcitriol increases total K^+^ currents in ventricular myocytes. **(A)** Superimposed current records of total K^+^ current (I_total_) for 1 myocyte treated with vehicle and 1 myocyte treated with 10 nM calcitriol. **(B)** Current-voltage curves for I_total_ density obtained in myocytes treated with vehicle or calcitriol. **(C)** Time course of the effect of calcitriol on I_total_ by perforated patch-clamp recordings. The black points in the figure are peak outward currents recorded at +50 mV every 10 s. Horizontal line indicates exposure to calcitriol 10 nM and washout. Upper panel shows I_total_ traces at +50 mV corresponding to time 0 min **(a)**, 9 min after calcitriol perfusion **(b),** and ∼10 min after washout **(c)**. Data are expressed as means ± SEM. C_m_ = membrane capacitance in pF. ^∗∗∗^*P* < 0.001; ^∗∗^*P* < 0.01; ^∗^*P* < 0.05.

For the experimental setup, the main K^+^ currents responsible for the AP repolarization phase in the mouse were recorded in ventricular myocytes treated either with vehicle or 10 nM calcitriol for 15–30 min. In the adult mouse ventricle, outward K^+^ currents involved in cardiac repolarization comprise three components that can be distinguished by their specific time and voltage dependency and also their sensitivity to pharmacological agents. **Figure 1** illustrates the protocol used to identify the three components: the fast transient outward current (I_tof_), the ultrarapid delayed rectifier K^+^ current (I_kur_) and the non-inactivating steady-state outward current (I_ss_). As a first step the total K^+^ currents were recorded by applying 300 ms depolarizing steps from −50 to +50 mV from a holding potential of −80 mV (**Figure 1A**). Then, I_tof_ was inactivated by applying a short prepulse (100 ms step at −30 mV) from a holding potential of −80 mV, followed by depolarizing steps from −50 to +50 mV. I_tof_ was then calculated by substracting the current recording with (**Figure 1A**) and without (**Figure 1B**) the inactivating prepulse. The current that remains after I_tof_ was inactivated, is composed of I_ss_ and I_Kur_. To separate both currents, a low concentration of 4-aminopyridine (4-AP, 250 μM), which selectively blocks I_kur_, was applied to cells that were prepulsed to inactivate I_tof_. The current remaining after this protocol is I_ss_ (**Figure 1C**), and I_kur_ can then be obtained by subtracting the current in the absence (**Figure 1B**) and in the presence (**Figure 1C**) of 4-AP. **Figure 2A** shows representatives recordings of total K^+^ currents obtained in 1 myocyte treated with vehicle and in 1 myocyte treated with 10 nM calcitriol. **Figure 2B** shows mean current-voltage (IV) curves for I_total_ density. Myocytes treated with calcitriol showed higher values of I_total_ density than those treated with vehicle (at +50 mV, vehicle 38.2 ± 2.2 pA/pF, *n* = 27; calcitriol 50.5 ± 3.1 pA/pF, *n* = 30; *P* < 0.01). In another group of experiments we tested the effect of calcitriol on total K^+^ currents by analyzing the time dependence of the effect in continuous recordings. For that, we used the perforated patch-clamp technique which preserves the cell from loss of some cytosolic second messengers due to intracellular dialysis that occurs during prolonged whole-cell recordings. **Figure 2C** (upper panel) shows representative traces of I_total_ corresponding to points a, b, and c on the graph below (lower panel). Peak I_total_ was elicited by depolarizing pulses from a holding potential of −80 to +50 mV every 10 s over 19 min. Application of calcitriol induced a rapid increase in I_total_, which reached a maximum effect at 9–10 min; then, the cardiomyocyte was perfused with calcitriol-free solution, which led to a progressive decrease in I_total_ toward control values after ∼10 min of washout. Similar results were obtained in three other cardiomyocytes.

**FIGURE 3:**
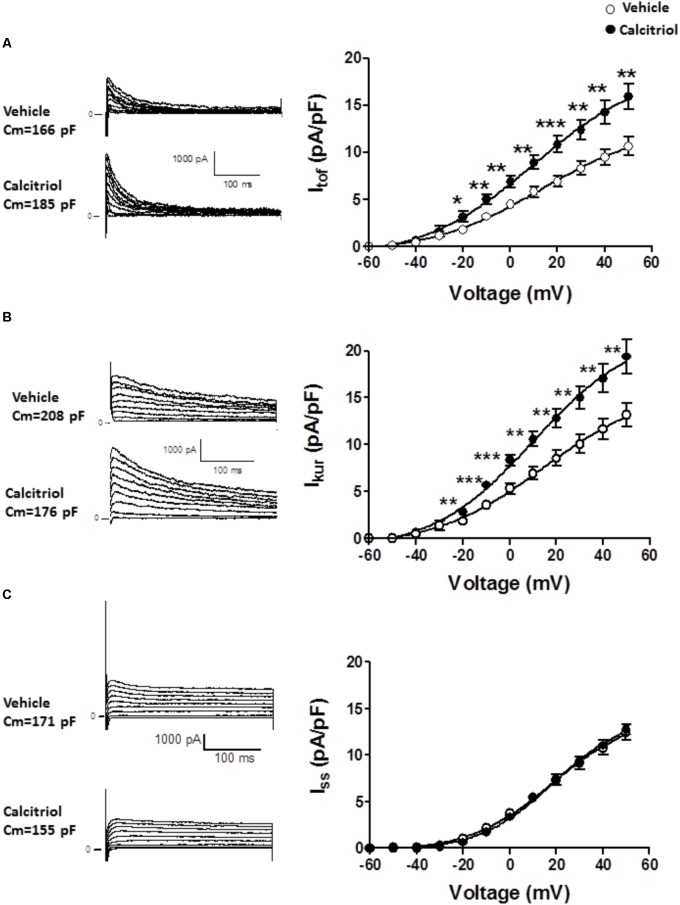
The fast transient outward K^+^ current (I_tof_) and the ultrarapid delayed rectifier K^+^ current (I_kur_) are significantly increased in calcitriol-treated myocytes. **(A)** *Left panel*: representative I_tof_ traces for 1 myocyte treated with vehicle and 1 myocyte treated with 10 nM calcitriol. *Right panel*: I/V curves for I_tof_ density obtained in myocytes treated with vehicle or with 10 nM calcitriol. **(B)** *Left panel*: representative I_kur_ traces for 1 myocyte treated with vehicle and 1 myocyte treated with calcitriol. *Right panel*: I/V curves for I_kur_ density obtained in myocytes treated with vehicle or calcitriol. **(C)** *Left panel*: representative I_ss_ traces for 1 myocyte treated with vehicle and 1 myocyte treated with calcitriol. *Right panel*: IV curves for I_ss_x densities obtained in myocytes treated with vehicle or with calcitri. Data are expressed as means ± SEM. C_m_ = membrane capacitance in pF. ^∗∗∗^*P* < 0.001; ^∗∗^*P* < 0.01; ^∗^*P* < 0.05.

**Figure 3A** (left panel) shows representative recordings of I_tof_ obtained in 1 myocyte treated with vehicle and in 1 myocyte treated with 10 nM calcitriol a, and **Figure 3A** (right panel) shows (IV) curves for I_tof_ density. Myocytes treated with calcitriol showed higher values of I_tof_ density than those treated with vehicle (at +50 mV, vehicle: 10.6. ± 0.9 pA/pF, *n* = 23; calcitriol: 15.9 ± 1.4 pA/pF, *n* = 28; *P* < 0.01). **Figure 3B** (left panel) illustrates representative recordings of I_kur_ obtained in 1 myocyte treated with vehicle and in 1 myocyte treated with 10 nM calcitriol l), **Figure 3B** (right panel) shows mean IV curves for I_kur_ density). Myocytes treated with calcitriol showed higher values of I_kur_ density than those treated with vehicle (at +50 mV, vehicle: 13.2 ± 1.2 pA/pF, *n* = 25; calcitriol: 19.4 ± 1.8 pA/pF, *n* = 24; *P* < 0.01). Representative recordings of I_ss_ obtained in 1 myocyte treated with vehicle and in 1 myocyte treated with 10 nM calcitriol are shown in **Figure 3C** left panel, and mean IV curves for I_ss_ density are shown in **Figure 3C** right panel. I_ss_ values in myocytes treated with vehicle (at +50 mV, 12.4 ± 0.8 pA/pF, *n* = 12) were similar to those of myocytes treated with calcitriol (at +50 mV, 12.8 ± 0.5 pA/pF, *n* = 26).

### Calcitriol Treatment Does Not Modify Action Potentials Duration

**FIGURE 4:**
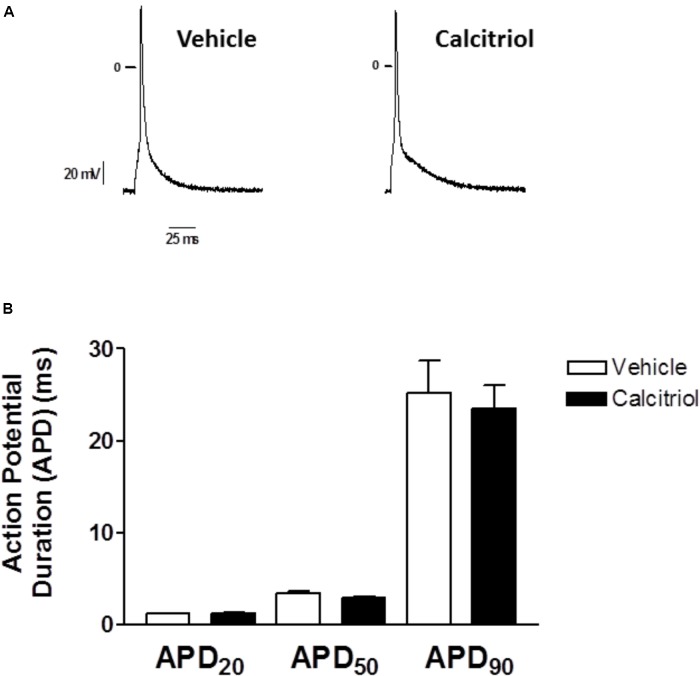
Effects of calcitriol on Action Potentials Duration **(A)** Representative traces of action potentials recorded in 1 myocyte treated with vehicle and in 1 myocyte treated with 10 nM calcitriol. **(B)** Bar graph showing mean action potential duration (APD) values measured at 20, 50, and 90% of repolarization (APD20, APD50, and APD90, respectively). Data are expressed as means ± SEM.

Since an increase in outward current can modulate repolarization, we carried out current clamp experiments to measure AP duration (APD) in myocytes treated with vehicle or calcitriol. Representative traces of APs recorded in 1 ventricular myocyte treated with vehicle (left) and in 1 myocyte treated with calcitriol (right) are shown in **Figure 4A**. Mean values of APD measured at 20, 50, and 90% of repolarization obtained in ventricular myocytes treated with vehicle or calcitriol are illustrated in **Figure 4B**. Values of APD were similar in both groups: APD20, vehicle 1.2 ± 0.6 ms vs. calcitriol 1.3 ± 0.1 ms; APD50, vehicle 3.4 ± 0.3 ms vs. calcitriol 2.9 ± 0.2 ms; APD90, vehicle 25.2 ± 3.5 ms vs. calcitriol 23.5 ± 2.5 ms (*n* = 10 cells treated with vehicle or calcitriol). In addition, no differences were observed in resting potential (RP) or AP amplitude in myocytes treated with vehicle or with calcitriol (−78.3 ± 1.9 mV vs. −77.4 ± 1.4 mV for RP and 117.7 ± 3.7 mV vs. 120.7 ± 3.6 mV for AP amplitude, vehicle vs. calcitriol-treated cells, respectively).

### Vitamin D Receptor Is Involved in the Effect of Calcitriol on Outward K^+^ Currents

**FIGURE 5:**
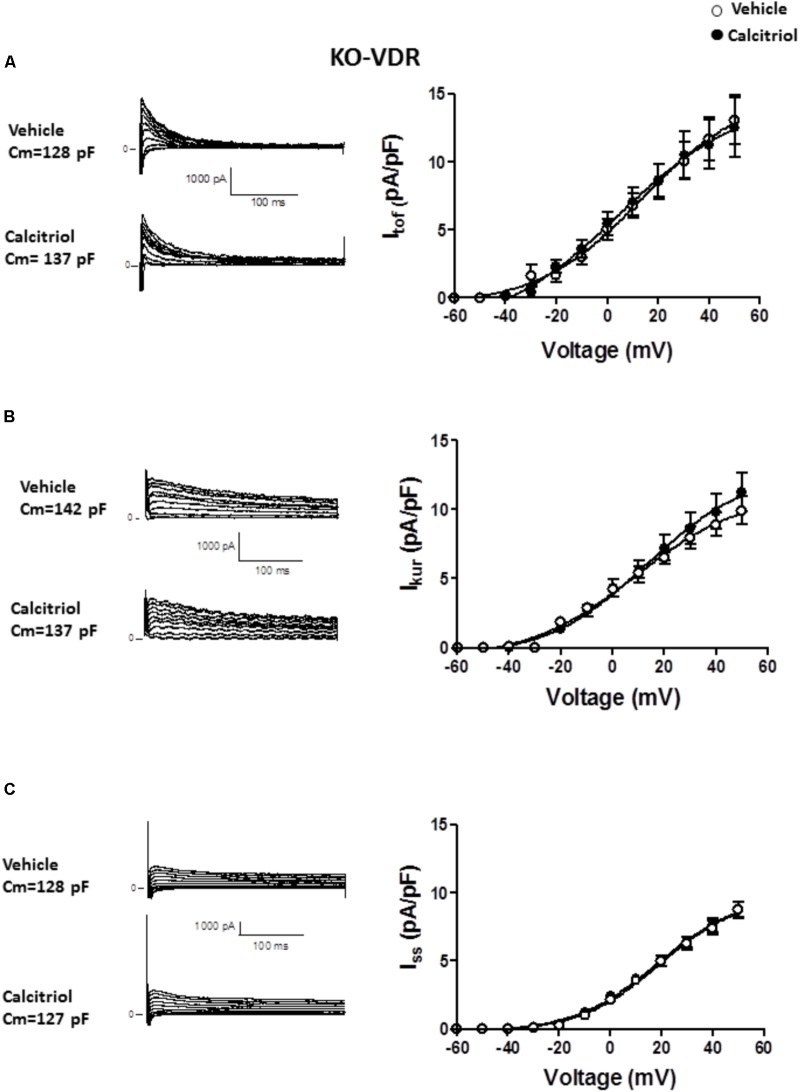
Effect of calcitriol on I_tof_ and I_kur_ involves binding to the vitamin D receptor. **(A)** *Left panel*: representative traces of I_tof_ obtained in 1 VDR-KO myocyte treated with vehicle and in 1 VDR-KO myocyte treated with 10 nM calcitriol. *Right panel*: I/V curves for I_tof_ density obtained in VDR-KO myocytes treated with vehicle or calcitriol. **(B)** *Left panel*: representative traces of I_kur_ obtained in 1 VDR-KO myocyte treated with vehicle and in 1 VDR-KO myocyte treated with 10 nM calcitriol. *Right panel*: I/V curves for I_kur_ density obtained in VDR-KO myocytes treated with vehicle or calcitriol. **(C)** *Left panel*: representative traces of I_ss_ obtained in 1 VDR-KO myocyte treated with vehicle and in 1 VDR-KO myocyte treated with 10 nM calcitriol. *Right panel*: I/V curves for I_ss_ density obtained in VDR-KO myocytes treated with vehicle or calcitriol. Data are expressed as means ± SEM. C_m_ = membrane capacitance in pF.

To examine the participation of VDR in the effect of calcitriol on I_tof_ and I_kur_, ventricular myocytes were isolated from VDR-KO mice and treated with vehicle or 10 nM calcitriol for 15–30 min. Representative recordings of I_tof_, obtained in 1 VDR-KO myocyte treated with vehicle or 10 nM calcitriol are shown in **Figure 5A** left panel, and mean IV curves for I_tof_ density are shown in **Figure 5A** right panel. In VDR-deficient myocytes, calcitriol was unable to increase I_tof_ density when compared with vehicle (at +50 mV, vehicle: 13.1 ± 1.8 pA/pF, *n* = 10; calcitriol: 12.5 ± 2.2 pA/pF, *n* = 6). Representative recordings of I_kur_, obtained in 1 VDR-KO myocyte treated with vehicle and in 1 VDR-KO myocyte treated with 10 nM calcitriol are shown in **Figure 5B** (left panel), and mean IV curves for I_kur_ density are shown in **Figure 5B** right panel. In the absence of VDR, the mean I_kur_ density was similar between myocytes treated with vehicle or calcitriol (at +50 mV, vehicle: 9.9 ± 1.0 pA/pF, *n* = 6; calcitriol: 11.2 ± 1.4 pA/pF, *n* = 9). Representative recordings of I_ss_, obtained in 1 VDR-KO myocyte treated with vehicle and in 1 VDR-KO myocyte treated with 10 nM calcitriol are shown in **Figure 5C** (left panel), and mean IV curves for I_ss_ density in VDR-KO myocytes treated with vehicle or calcitriol are shown in **Figure 5C** right panel. Results showed that calcitriol had no effect on I_ss_ density in VDR-KO myocytes (at +50 mV, vehicle: 8.7 ± 0.6 pA/pF, *n* = 13; calcitriol: 8.8 ± 0.6 pA/pF, *n* = 10).

### Akt Mediates the Stimulatory Effect of Calcitriol on I_tof_ and I_kur_

**FIGURE 6:**
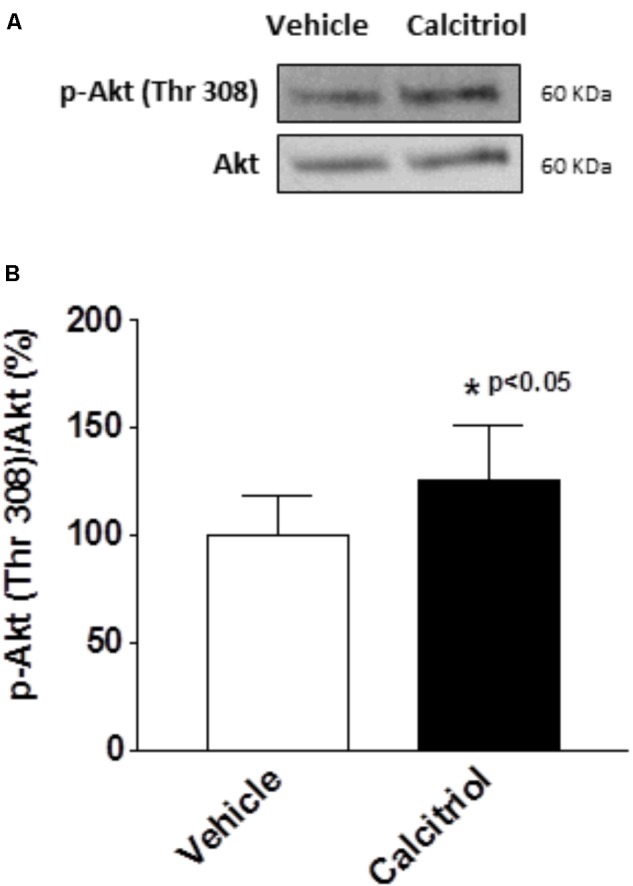
Calcitriol activates the Akt pathway in ventricular myocytes. Ventricular myocytes were exposed to vehicle or calcitriol 10 nM for 15 min. **(A)** Representative western blot and **(B)** average ratio of protein levels expressed as the percentage of phospho (P)-Akt normalized to total Akt. Data are expressed as mean ± SEM. ^∗^*P* < 0.05.

**FIGURE 7:**
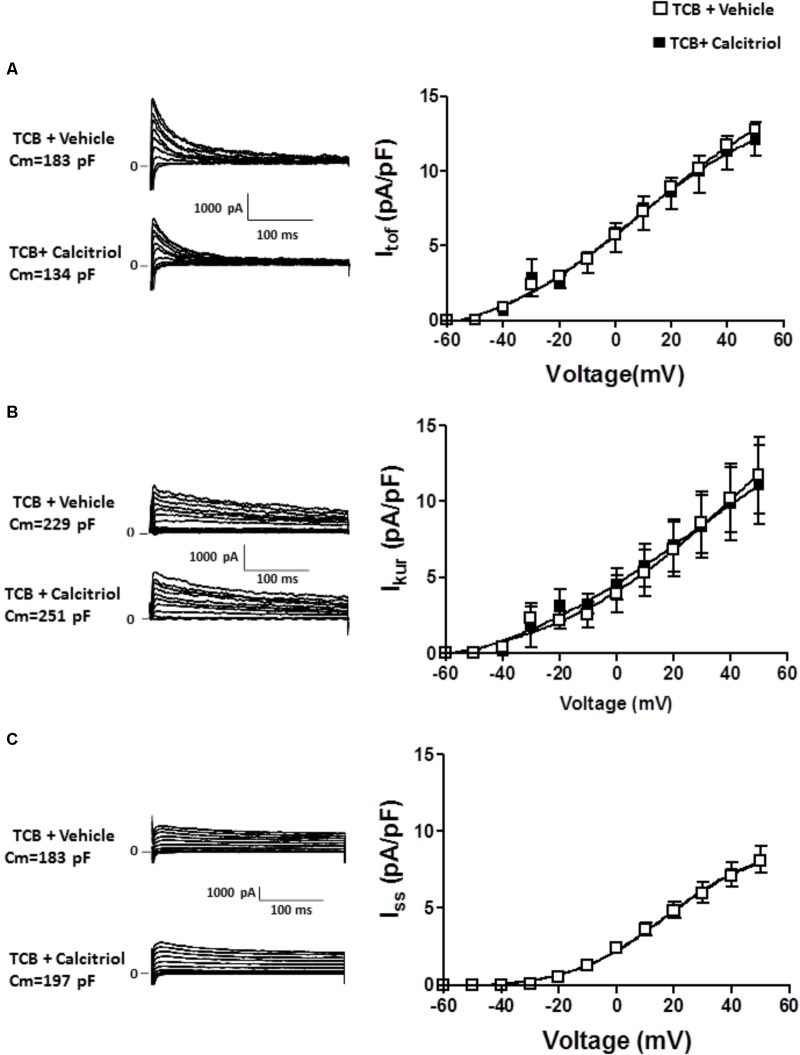
Inhibition of Akt signaling prevents the increase in I_tof_ and I_kur_ densities induced by calcitriol. **(A)** *Left panel*: I_tof_ traces obtained in 1 myocyte treated with 1 μM triciribine and in 1 myocyte treated with 1 μM triciribine plus 10 nM calcitriol. *Right panel*: I/V curves for I_tof_ density obtained in myocytes treated with 1 μM triciribine or with triciribine plus 10 nM calcitriol. **(B)** *Left panel*: I_kur_ traces obtained in one myocyte treated with 1 μM triciribine and in 1 myocyte treated with 1 μM triciribine plus 10 nM calcitriol. *Right panel*: I/V curves for I_kur_ density obtained in myocytes treated with 1 μM triciribine or with triciribine plus 10 nM calcitriol. **(C)** *Left panel*: I_ss_ traces obtained in 1 myocyte treated with 1 μM triciribine and in 1 myocyte treated with 1 μM triciribine plus 10 nM calcitriol. *Right panel*: I/V curves for I_ss_ density obtained in myocytes treated with 1 μM triciribine or with triciribine plus 10 nM calcitriol. Data are expressed as means ± SEM. C_m_, membrane capacitance; TCB, triciribine.

It has been postulated that the activation of the PI3K pathway can modulate cardiac K^+^ channels (Yang et al., 2012; Gomez-Hurtado et al., 2014). To explore the implication of PI3K signaling in the calcitriol-induced increase of I_tof_ and I_kur_ densities, we questioned whether calcitriol activates Akt, a known target of PI3K, in ventricular myocytes (Shioi et al., 2002; McMullen et al., 2003). We observed a significant activation of Akt (measured as phosphorylation) after 15 min of calcitriol treatment (**Figures 6A,B**). We therefore measured I_tof_, I_kur_, and I_ss_ in myocytes pretreated with 1 μM of the Akt inhibitor triciribine. Representative traces obtained in myocytes pretreated with triciribine and then treated with vehicle or calcitriol are shown in **Figures 7A–C**, respectively (left panels), and mean IV curves for I_tof_, I_kur_ and I_ss_ densities in both situations are shown in the corresponding right panels. The results showed that treatment with triciribine prevented the stimulatory effect of calcitriol on I_tof_ and I_Kur_.

## Discussion

Cardiac APs are the consequence of the coordinated functioning of inward and outward current flowing through specialized proteins (ionic channels) that open and close in a voltage- and time-dependent manner allowing rhythmic and effective cardiac contractions (Gaborit et al., 2007). It is well known that K^+^ current density is reduced during heart remodeling under various pathological conditions including left ventricular hypetrophy and HF. This process is associated with prolonged QT interval, increased risk of malignant arrhythmias and sudden cardiac death (Ravens and Cerbai, 2008). Here, we tested the hypothesis that calcitriol might have a cardioprotective effect by increasing K^+^ currents, thereby circumventing inappropriate prolongation” of the APD during pathology. In mice, the repolarization phase of the AP is determined principally by the outward currents I_tof_ and I_kur_, which account for the remarkably abbreviated APs and the absence of a clear plateau phase in this species. We found that calcitriol significantly increased I_tof_ and I_kur_ densities. In apparent contrast, we found that the APD was similar in myocytes treated or not with calcitriol. The lack of the effect of increased K^+^ currents on the APD in calcitriol-treated myocytes indicates that calcitriol might affect other ionic currents that influence the APD. In this sense, we previously demonstrated that calcitriol can increase L-type calcium current (I_CaL_) density in ventricular myocytes (Tamayo et al., 2017). The increase of I_CaL_ might counterbalance the effect of increased outward K^+^ currents in calcitriol-treated myocytes, preserving the APD. Calcitriol can induce genomic (transcription-dependent) and non-genomic (transcription-independent) actions. Indeed, there is a general agreement in the idea that calcitriol effects observed within minutes (such as those described in the present study) involve only non-genomic effects mediated by extra-nuclear mechanisms (Mizwicki and Norman, 2009). Most calcitriol actions are mediated by VDR. Classic nuclear VDR is required for genomic effects, whereas non-genomic actions are thought to be mediated by a fraction of VDR located in the cytosol or at the plasma membrane (Norman et al., 2001a,b). In this regards, *in silico* studies have been useful to determine exactly how calcitriol binds to VDR to regulate both genomic and non-genomic responses. An alternative ligand-binding pocket has been identified in VDR that specifically mediates non-genomic actions and that partially overlaps the genomic pocket described in the experimentally determined x-ray structure (Mizwicki et al., 2004). In the present study, we used VDR-KO mice to determine whether the effect of calcitriol on K^+^ currents in cardiomyocytes involves VDR. Our results clearly show that the absence of VDR abolished all changes in K^+^ currents induced by the acute administration of calcitriol, demonstrating that these effects are VDR-dependent. Consistent with this, we have previously described that calcitriol exerts VDR-dependent non-genomic effects on Ca^2+^ current and PKA signaling in cardiomyocytes (Tamayo et al., 2017), which is in line with the findings by Zanello and Norman (2006) showing that a functional VDR is required for the rapid increase in Cl- current and for the shift in the peak activation of I_CaL_ current induced by calcitriol in osteoblasts. As VDR-KO mice do not express VDR, this model does not allow us to discriminate precisely which VDR location mediates these observed effects. We are, however, inclined to favor a non-genomic action mediated by membrane VDR due to the short time required (15–30 min) and the fact that VDR has been reported to be expressed in the tubular system of ventricular myocytes (Tishkoff et al., 2008), a region where voltage-gated calcium and potassium channels are predominantly localized (Takeuchi et al., 2000; Brette and Orchard, 2003). On the other hand, protein disulfide isomerase family A member 3 (PDIA3) has been proposed as a membrane receptor for calcitriol that mediates some of its non-genomic effects in chondrocytes, osteoblasts and other cell types (Boyan et al., 2003; Doroudi et al., 2014a,b). Intriguingly, both PDIA3 and VDR are located in caveolae in many tissues and cell types, and an interaction between them has been observed, suggesting that VDR and PDIA3 might both be required for some non-genomic actions of calcitriol (Sequeira et al., 2012; Chen et al., 2013). As the effects of calcitriol on K^+^ current in cardiomyocytes are completely abolished in VDR-KO cells we would argue that PDIA3 is not implicated in the described effect, although clearly experiments with PDIA3-KO cells would be necessary to completely discard the possibility that both VDR and PDIA3 are involved.

Several reports have shown that calcitriol binding to plasma membrane VDR induces rapid non-transcriptional responses through the activation of different signaling pathways including PI3K/Akt (Buitrago et al., 2012; Salles et al., 2013). This pathway is widely expressed in eukaryotes and is known to play key roles in growth, differentiation, proliferation, and survival (Cantley, 2002). In the heart, PI3K/Akt activation has been related to both physiological cardiac enlargement and protection (McMullen et al., 2003; Weeks et al., 2012). Moreover, it is believed that the PI3K/Akt pathway might regulate cardiac ion channels and arrhythmogenesis (Ballou et al., 2015). Our results show that calcitriol activates Akt in adult ventricular myocytes, and its pharmacological inhibition prevents the calcitriol-induced increase of I_tof_ and I_kur_ densities.

In sum, we demonstrate that calcitriol binding to VDR increases the density of the two main K^+^ currents responsible for the rapid repolarization of the AP in the mouse, I_tof_ and I_Kur_. Our study also supports a role of Akt signaling in this stimulatory effect.

## Author Contributions

MT performed most of the experiments and analyzed the data. LM-N isolated ventricular myocytes and performed most of the western blotting experiments. MP took part in the critical discussion and editing of the manuscript. ML provided VDR-KO mice. AV-B and NG-H helped with western blotting. ML and NG-H took part in the critical discussion and editing of the manuscript. MF-V designed the experiments, reviewed the data, and wrote the manuscript. CD performed some electrophysiological experiments, conceived the hypothesis, supervised the project, reviewed the data, and wrote the manuscript.

## Conflict of Interest Statement

The authors declare that the research was conducted in the absence of any commercial or financial relationships that could be construed as a potential conflict of interest.

## References

[B1] Ballou L. M. Lin R. Z. Cohen I. S. (2015). Control of cardiac repolarization by phosphoinositide 3-kinase signaling to ion channels. *Circ. Res.* 116 127–137. 10.1161/CIRCRESAHA.116.303975 25552692 PMC4283553

[B2] Boyan B. D. Sylvia V. L. McKinney N. Schwartz Z. (2003). Membrane actions of vitamin D metabolites 1alpha,25(OH)2D3 and 24R,25(OH)2D3 are retained in growth plate cartilage cells from vitamin D receptor knockout mice. *J. Cell. Biochem.* 90 1207–1223. 10.1002/jcb.10716 14635194

[B3] Brette F. Orchard C. (2003). T-tubule function in mammalian cardiac myocytes. *Circ. Res.* 92 1182–1192. 10.1161/01.RES.0000074908.17214.FD 12805236

[B4] Brouillette J. Clark R. B. Giles W. R. Fiset C. (2004). Functional properties of K+ currents in adult mouse ventricular myocytes. *J. Physiol.* 559 777–798. 10.1113/jphysiol.2004.063446 15272047 PMC1665169

[B5] Buitrago C. G. Arango N. S. Boland R. L. (2012). 1α,25(OH)2D3-dependent modulation of Akt in proliferating and differentiating C2C12 skeletal muscle cells. *J. Cell. Biochem.* 113 1170–1181. 10.1002/jcb.23444 22095470

[B6] Cantley L. C. (2002). The phosphoinositide 3-kinase pathway. *Science* 296 1655–1657. 10.1126/science.296.5573.1655 12040186

[B7] Chen J. Doroudi M. Cheung J. Grozier A. L. Schwartz Z. Boyan B. D. (2013). Plasma membrane Pdia3 and VDR interact to elicit rapid responses to 1α,25(OH)(2)D(3). *Cell. Signal.* 25 2362–2373. 10.1016/j.cellsig.2013.07.020 23896121

[B8] Delgado C. Ruiz-Hurtado G. Gomez-Hurtado N. Gonzalez-Ramos S. Rueda A. Benito G. (2015). NOD 1, a new player in cardiac function and calcium handling. *Cardiovasc. Res.* 106 375–386. 10.1093/cvr/cvv118 25824149

[B9] Doroudi M. Boyan B. D. Schwartz Z. (2014a). Rapid 1α,25(OH)_2_D_3_ membrane-mediated activation of Ca^2+^/calmodulin-dependent protein kinase II in growth plate chondrocytes requires Pdia3. PLAA and caveolae. *Connect. Tissue Res.* 55(Suppl. 1), 125–128. 25158196 10.3109/03008207.2014.923882

[B10] Doroudi M. Chen J. Boyan B. D. Schwartz Z. (2014b). New insights on membrane mediated effects of 1α,25-dihydroxy vitamin D3 signaling in the musculoskeletal system. *Steroids* 81 81–87. 10.1016/j.steroids.2013.10.019 24291576

[B11] Gaborit N. Le Bouter S. Szuts V. Varro A. Escande D. Nattel S. (2007). Regional and tissue specific transcript signatures of ion channel genes in the non-diseased human heart. *J. Physiol.* 582 675–693. 10.1113/jphysiol.2006.126714 17478540 PMC2075332

[B12] Gómez-Hurtado N. Domínguez-Rodríguez A. Mateo P. Fernández-Velasco M. Val-Blasco A. Aizpún R. (2017). Beneficial effects of leptin treatment in a setting of cardiac dysfunction induced by transverse aortic constriction in mouse. *J. Physiol.* 595 4227–4243. 10.1113/JP274030 28374413 PMC5491865

[B13] Gomez-Hurtado N. Fernandez-Velasco M. Fernandez-Alfonso M. Bosca L. Delgado C. (2014). Prolonged leptin treatment increases transient outward K+ current via upregulation of Kv4.2 and Kv4.3 channel subunits in adult rat ventricular myocytes. *Pflugers Arch.* 466 903–914. 10.1007/s00424-013-1348-3 24046152

[B14] Gotsman I. Shauer A. Zwas D. R. Hellman Y. Keren A. Lotan C. (2012). Vitamin D deficiency is a predictor of reduced survival in patients with heart failure; vitamin D supplementation improves outcome. *Eur. J. Heart Fail.* 14 357–366. 10.1093/eurjhf/hfr175 22308011

[B15] Hartupee J. Mann D. L. (2017). Neurohormonal activation in heart failure with reduced ejection fraction. *Nat. Rev. Cardiol.* 14 30–38. 10.1038/nrcardio.2016.163 27708278 PMC5286912

[B16] Heusch G. Libby P. Gersh B. Yellon D. Böhm M. Lopaschuk G. (2014). Cardiovascular remodelling in coronary artery disease and heart failure. *Lancet* 383 1933–1943. 10.1016/S0140-6736(14)60107-0 24831770 PMC4330973

[B17] Holick M. F. (2007). Vitamin D deficiency. *N. Engl. J. Med.* 357 266–281. 10.1056/NEJMra070553 17634462

[B18] Larriba M. J. González-Sancho J. M. Bonilla F. Muñoz A. (2014). Interaction of vitamin D with membrane-based signaling pathways. *Front. Physiol.* 5:60. 10.3389/fphys.2014.00060 24600406 PMC3927071

[B19] Lehnart S. E. Maier L. S. Hasenfuss G. (2009). Abnormalities of calcium metabolism and myocardial contractility depression in the failing heart. *Heart Fail. Rev.* 14 213–224. 10.1007/s10741-009-9146-x 19434491 PMC2772965

[B20] Li Y. C. Pirro A. E. Amling M. Delling G. Baron R. Bronson R. (1997). Targeted ablation of the vitamin D receptor: an animal model of vitamin D-dependent rickets type II with alopecia. *Proc. Natl. Acad. Sci. U.S.A.* 94 9831–9835. 10.1073/pnas.94.18.9831 9275211 PMC23277

[B21] Liu L. C. Voors A. A. van Veldhuisen D. J. van der Veer E. Belonje A. M. Szymanski M. K. (2011). Vitamin D status and outcomes in heart failure patients. *Eur. J. Heart Fail.* 13 619–625. 10.1093/eurjhf/hfr032 21543375

[B22] Ma Y. Yu W. D. Kong R. X. Trump D. L. Johnson C. S. (2006). Role of nongenomic activation of phosphatidylinositol 3-kinase/Akt and mitogen-activated protein kinase/extracellular signal-regulated kinase kinase/extracellular signal-regulated kinase 1/2 pathways in 1,25D3-mediated apoptosis in squamous cell carcinoma cells. *Cancer Res.* 66 8131–8138. 10.1158/0008-5472.CAN-06-1333 16912191

[B23] McMullen J. R. Shioi T. Zhang L. Tarnavski O. Sherwood M. C. Kang P. M. (2003). Phosphoinositide 3-kinase(p110alpha) plays a critical role for the induction of physiological, but not pathological, cardiac hypertrophy. *Proc. Natl. Acad. Sci. U.S.A.* 100 12355–12360. 10.1073/pnas.1934654100 14507992 PMC218762

[B24] Menegaz D. Mizwicki M. T. Barrientos-Duran A. Chen N. Henry H. L. Norman A. W. (2011). Vitamin D receptor (VDR) regulation of voltage-gated chloride channels by ligands preferring a VDR-alternative pocket (VDR-AP). *Mol. Endocrinol.* 25 1289–1300. 10.1210/me.2010-0442 21659475 PMC3146250

[B25] Mizwicki M. T. Keidel D. Bula C. M. Bishop J. E. Zanello L. P. Wurtz J. M. (2004). Identification of an alternative ligand-binding pocket in the nuclear vitamin D receptor and its functional importance in 1alpha,25(OH)2-vitamin D3 signaling. *Proc. Natl. Acad. Sci. U.S.A.* 101 12876–12881. 10.1073/pnas.0403606101 15326291 PMC516488

[B26] Mizwicki M. T. Norman A. W. (2009). The vitamin D sterol-vitamin D receptor ensemble model offers unique insights into both genomic and rapid-response signaling. *Sci. Signal.* 2:re4. 10.1126/scisignal.275re4 19531804

[B27] Nass R. D. Aiba T. Tomaselli G. F. Akar F. G. (2008). Mechanisms of disease: ion channel remodeling in the failing ventricle. *Nat. Clin. Pract. Cardiovasc. Med.* 5 196–207. 10.1038/ncpcardio1130 18317475

[B28] Nattel S. Maguy A. Le Bouter S. Yeh Y. H. (2007). Arrhythmogenic ion-channel remodeling in the heart: heart failure, myocardial infarction, and atrial fibrillation. *Physiol. Rev.* 87 425–456. 10.1152/physrev.00014.2006 17429037

[B29] Norman A. W. Henry H. L. Bishop J. E. Song X. D. Bula C. Okamura W. H. (2001a). Different shapes of the steroid hormone 1alpha,25(OH)(2)-vitamin D(3) act as agonists for two different receptors in the vitamin D endocrine system to mediate genomic and rapid responses. *Steroids* 66 147–158. 11179722 10.1016/s0039-128x(00)00165-3

[B30] Norman A. W. Ishizuka S. Okamura W. H. (2001b). Ligands for the vitamin D endocrine system: different shapes function as agonists and antagonists for genomic and rapid response receptors or as a ligand for the plasma vitamin D binding protein. *J. Steroid Biochem. Mol. Biol.* 76 49–59. 10.1016/S0960-0760(00)00145-X 11384863

[B31] Norman P. E. Powell J. T. (2014). Vitamin D and cardiovascular disease. *Circ. Res.* 114 379–393. 10.1161/CIRCRESAHA.113.301241 24436433

[B32] Ravens U. Cerbai E. (2008). Role of potassium currents in cardiac arrhythmias. *Europace* 10 1133–1137. 10.1093/europace/eun193 18653669

[B33] Salles J. Chanet A. Giraudet C. Patrac V. Pierre P. Jourdan M. (2013). 1,25(OH)2-vitamin D3 enhances the stimulating effect of leucine and insulin on protein synthesis rate through Akt/PKB and mTOR mediated pathways in murine C2C12 skeletal myotubes. *Mol. Nutr. Food Res.* 57 2137–2146. 10.1002/mnfr.201300074 23929734

[B34] Sequeira V. B. Rybchyn M. S. Tongkao-On W. Gordon-Thomson C. Malloy P. J. Nemere I. (2012). The role of the vitamin D receptor and ERp57 in photoprotection by 1α,25-dihydroxyvitamin D3. *Mol. Endocrinol.* 26 574–582. 10.1210/me.2011-1161 22322599 PMC3327356

[B35] Shioi T. McMullen J. R. Kang P. M. Douglas P. S. Obata T. Franke T. F. (2002). Akt/protein kinase B promotes organ growth in transgenic mice. *Mol. Cell. Biol.* 22 2799–2809. 10.1128/MCB.22.8.2799-2809.2002 11909972 PMC133704

[B36] Takeuchi S. Takagishi Y. Yasui K. Murata Y. Toyama J. Kodama I. (2000). Voltage-gated K(+)Channel, Kv4.2, localizes predominantly to the transverse-axial tubular system of the rat myocyte. *J. Mol. Cell. Cardiol.* 32 1361–1369. 10.1006/jmcc.2000.1172 10860776

[B37] Tamayo M. Manzanares E. Bas M. Martin-Nunes L. Val-Blasco A. Larriba M. J. (2017). Calcitriol (1,25-dihydroxyvitamin D3) increases L-type calcium current via protein kinase A signaling and modulates calcium cycling and contractility in isolated mouse ventricular myocytes. *Heart Rhythm* 14 432–439. 10.1016/j.hrthm.2016.12.013 27989685

[B38] Tishkoff D. X. Nibbelink K. A. Holmberg K. H. Dandu L. Simpson R. U. (2008). Functional vitamin D receptor (VDR) in the t-tubules of cardiac myocytes: VDR knockout cardiomyocyte contractility. *Endocrinology* 149 558–564. 10.1210/en.2007-0805 17974622 PMC2219302

[B39] Trépanier-Boulay V. St-Michel C. Tremblay A. Fiset C. (2001). Gender-based differences in cardiac repolarization in mouse ventricle. *Circ. Res.* 89 437–444. 10.1161/hh1701.095644 11532905

[B40] Vertino A. M. Bula C. M. Chen J. R. Almeida M. Han L. Bellido T. (2005). Nongenotropic, anti-apoptotic signaling of 1alpha,25(OH)2-vitamin D3 and analogs through the ligand binding domain of the vitamin D receptor in osteoblasts and osteocytes. Mediation by Src, phosphatidylinositol 3-, and JNK kinases. *J. Biol. Chem.* 280 14130–14137. 10.1074/jbc.M410720200 15671029

[B41] Weeks K. L. Gao X. Du X. J. Boey E. J. Matsumoto A. Bernardo B. C. (2012). Phosphoinositide 3-kinase p110α is a master regulator of exercise-induced cardioprotection and PI3K gene therapy rescues cardiac dysfunction. *Circ. Heart Fail.* 5 523–534. 10.1161/CIRCHEARTFAILURE.112.966622 22705768

[B42] Witte K. K. Byrom R. Gierula J. Paton M. F. Jamil H. A. Lowry J. E. (2016). Effects of Vitamin D on cardiac function in patients with chronic HF: the vindicate Study. *J. Am. Coll. Cardiol.* 67 2593–2603. 10.1016/j.jacc.2016.03.508 27058906 PMC4893154

[B43] Yang K. C. Jay P. Y. McMullen J. R. Nerbonne J. M. (2012). Enhanced cardiac PI3Kα signalling mitigates arrhythmogenic electrical remodelling in pathological hypertrophy and heart failure. *Cardiovasc. Res.* 93 252–262. 10.1093/cvr/cvr283 22038742 PMC3258651

[B44] Zanatta L. Goulart P. B. Gonçalves R. Pierozan P. Winkelmann-Duarte E. C. Woehl V. M. (2012). 1α,25-dihydroxyvitamin D(3) mechanism of action: modulation of L-type calcium channels leading to calcium uptake and intermediate filament phosphorylation in cerebral cortex of young rats. *Biochim. Biophys. Acta* 1823 1708–1719. 10.1016/j.bbamcr.2012.06.023 22743040

[B45] Zanello L. P. Norman A. (2006). 1alpha,25(OH)2 vitamin D3 actions on ion channels in osteoblasts. *Steroids* 71 291–297. 10.1016/j.steroids.2005.09.012 16457860

[B46] Zhang X. Zanello L. P. (2008). Vitamin D receptor-dependent 1 alpha,25(OH)2 vitamin D3-induced anti-apoptotic PI3K/AKT signaling in osteoblasts. *J. Bone Miner. Res.* 23 1238–1248. 10.1359/jbmr.080326 18410228 PMC2680173

[B47] Zhang Y. Zhang J. Studzinski G. P. (2006). AKT pathway is activated by 1, 25-dihydroxyvitamin D3 and participates in its anti-apoptotic effect and cell cycle control in differentiating HL60 cells. *Cell Cycle* 5 447–451. 10.4161/cc.5.4.2467 16479173

